# *Kaustia mangrovi* gen. nov., sp. nov. isolated from Red Sea mangrove sediments belongs to the recently proposed *Parvibaculaceae* family within the order Rhizobiales

**DOI:** 10.1099/ijsem.0.004806

**Published:** 2021-05-17

**Authors:** Fatmah O. Sefrji, Ramona Marasco, Grégoire Michoud, Kholoud A. Seferji, Giuseppe Merlino, Daniele Daffonchio

**Affiliations:** ^1^​ Biological and Environmental Sciences and Engineering Division (BESE), Red Sea Research Center (RSRC), King Abdullah University of Science and Technology (KAUST), Thuwal, Saudi Arabia

**Keywords:** *Kaustia*, mangrove, Red Sea sediment, osmoadaptation, *Rhizobiales*, cultivation, diffusion chamber

## Abstract

We isolated a novel strain, R1DC25^T^, described as *Kaustia mangrovi* gen. nov. sp. nov. from the sediments of a mangrove forest on the coast of the Red Sea in Saudi Arabia. This isolate is a moderately halophilic, aerobic/facultatively anaerobic Gram-stain-negative bacterium showing optimum growth at between 30 and 40 °C, at a pH of 8.5 and with 3–5 % NaCl. The genome of R1DC25^T^ comprises a circular chromosome that is 4 630 536 bp in length, with a DNA G+C content of 67.3 mol%. Phylogenetic analyses based on the 16S rRNA gene sequence and whole-genome multilocus sequence analysis of 120 concatenated single-copy genes revealed that R1DC25^T^ represents a distinct lineage within the family *Parvibaculaceae* in the order *Rhizobiales* within the class *Alphaproteobacteria *. R1DC25^T^ showing 95.8, 95.3 and 94.5 % 16S rRNA gene sequence identity with *Rhodoligotrophos appendicifer *, *Rhodoligotrophos jinshengii* and *Rhodoligotrophos defluvii *, respectively. The predominant quinone was Q-10, and the polar lipids were phosphatidylglycerol, phosphatidylcholine, diphosphatidylglycerol, as well as several distinct aminolipids and lipids. The predominant cellular fatty acids were C_19 : 0_ cyclo *ω*8*c*, a combination of C_18 : 1_ *ω*7*c* and/or C_18 : 1_ *ω*6*c* and C_16 : 0_. On the basis of the differences in the phenotypic, physiological and biochemical characteristics from its known relatives and the results of our phylogenetic analyses, R1DC25^T^ (=KCTC 72348^T^;=JCM 33619^T^;=NCCB 100699^T^) is proposed to represent a novel species in a novel genus, and we propose the name *Kaustia mangrovi* gen. nov., sp. nov. (*Kaustia,* subjective name derived from the abbreviation KAUST for King Abdullah University of Science and Technology; *mangrovi*, of a mangrove).

## Introduction

Recent developments in technology have led to significant increases in our understanding of the extent of microbial diversity. Many species have been described using omics-based approaches, but a huge knowledge gap still exists between the genomic potential assessment and function assignment to genes and proteins [1]. By cultivating species, researchers can expand the available data about microbes and their genomes, even for those species that are not easily detected by molecular methods due to their scarcity in the environment, i.e. they are members of the rare biosphere [2]. The isolation and characterization of novel microbes from conventional and overlooked ecosystems remain a cornerstone of research in microbiology [3]. ‘Hidden’ microbial strains can sometimes be detected using alternative cultivation strategies, such as diffusion chambers (DCs) [4, 5]. In the present study, we used this novel approach to investigate the microbial diversity of the subtropical mangrove sediments in the arid environment of the coast of the Red Sea, an unexplored natural ecosystem. Mangrove forests are highly productive ecosystems and are widespread in tropical and subtropical coastlines, with a coverage of 60–70 % [6–9]. Mangroves represent unique ecological niches as they host a diverse variety of microorganisms [10–12]. It has been estimated that the bacteria in mangrove sediments constitute up to 80 % of the total living biomass of these ecosystems [13–16] and play a key role in their functioning [12]. However, relatively few studies have focused on the exploration and characterization of the microbial diversity of mangrove sediments [7, 10, 11, 13, 14, 17–27] and the cultivable fraction of microbes therein [28–34]. The cultivable bacterial strains obtained from mangrove sediments include members of well-characterized genera, such as *Bacillus *, *Halobacillus *, *Microbacterium *, *Novosphingobium *, *Paracoccus *, *Streptomyces *, *Thalassotalea* and *Vibrio* [30, 33, 35–39], and several novel genera, including *Acidimangrovimonas *, *Mangroviflexus *, *Mangrovibacterium *, *Marisediminitalea *, *Mangrovicoccus *, *Mangrovitalea *, *Mangrovimonas* and *Zhengella* [32, 34, 40–45]; these data clearly confirm the untapped diversity harboured by mangrove sediments. In this research, we used an inoculum of sediments collected from the mangrove forest of *Avicennia marina* in the Ibn-Sina Research Station, King Abdullah University of Science and Technology (KAUST), Saudi Arabia, located on the coast of the Red Sea. We isolated and described a novel culturable bacteria, *Kaustia mangrovi* gen. nov. sp. nov., belonging to the order *Rhizobiales *, class *Alphaproteobacteria *, family *Parvibaculaceae *. The order *Rhizobiales *, the taxonomy of which was recently modified [46], includes bacterial species that are ecologically important for soil, animals and plants [10, 47] and may also be important in the mangrove ecosystem. In this paper, we describe the genomic, physicochemical and metabolic features of R1DC25^T^.

## Isolation and habitat

R1DC25^T^ was isolated under aerobic conditions from Red Sea mangrove sediments using DC-based *in-situ* cultivation [5]. Mangrove sediments were collected in 2017 from the Ibn-Sina Research Station (22.34°N, 39.09°E) within the KAUST campus on the coast of the Red Sea of Saudi Arabia. The physiochemical characteristics of the sediments have been described by Booth *et al*. [10]. Dead mangrove leaves from the same area were also sampled. DCs comprise a 70 mm stainless-steel washer and two polycarbonate membranes with a diameter of 25 mm and a pore size of 0.03 µm (Osmonics). The DC was set up as previously described [4] and all procedures were performed in the sterile environment of a laminar flow hood. Using silicon glue, a sterile membrane filter (pore size, 0.03 µm) was glued to one side of the washer, covering the hole. Sediment and leaf extracts were obtained by mixing sediment or mangrove leaves with MilliQ water in a 1 : 10 ratio. The mixtures were autoclaved for 30 min, spun down for 10 min at 11 000 r.p.m. and then filter-sterilized using filters with a pore size of 0.22 µm. These extracts were stored at 4 °C for further use. The cultivation medium was prepared by diluting 1 g of fresh mangrove sediments with filtered sea water (FSW) and then mixing it with molten FSW-agar (1.5 %) and sediment or leaf extracts to a final concentration of 0.1 %, reaching a final dilution of 1×10^4^ g ml^−1^. Then, 3 ml of this mixture was used to fill the DCs, which were then sealed and incubated in an aquarium containing mangrove sediments and sea water to mimic the natural habitat. After 21 days, the DCs were taken from the aquarium, washed in pure MilliQ water and then opened under a laminar flow hood. The FSW-agar, along with its microbial biomass, was homogenized by passaging it through a sterile syringe with a 25-gauge needle and then diluted with molten FSW-agar and sediment or leaf extracts to obtain dilutions of 10^−4^ to 10^−6^ g ml^−1^ [47]. The final solution was poured into petri dishes and incubated at 37 °C for 7 days. Colonies showing growth were collected using glass Pasteur pipettes (337 mm diameter with long tip, Sigma-Aldrich) and subcultured in 0.1×Luria–Bertani (LB) agar supplemented with 0.1 % sediment or leaf extracts. Each isolate was restreaked three times to obtain a pure culture. The purity of the colonies was confirmed using a stereomicroscope (S8AP0; Leica). Bacterial cultures were maintained in marine broth (MB; peptone, 5 g; yeast extract, 1 g; C_6_H_5_FeO_7_, 0.1 g; NaCl, 19.45 g; MgCl_2_, 5.9 g; MgSO_4_, 3.24 g; CaCl_2_, 1.8 g; KCl, 0.55 g; NaHCO_3_, 0.16 g; KBr, 0.08 g; SrCl_2_, 34 mg; H_3_BO_3_, 22 mg; Na_2_SiO_3_, 4 mg; NaF, 2.4 mg; NH_4_NO_3_, 1.6 mg; Na_2_HPO_4_, 8 mg; final salinity using a refractometer, 4 %) at 37 °C. Bacterial culture stocks were further mixed with 30 % glycerol (v/v) and stored at −80 °C.

## Phylogenetic diversity of cultivable bacteria associated with mangroves

A total of 55 bacterial strains were isolated and their genomic DNA was extracted by boiling in 50 µl of 10 mM sterile Tris–HCl buffer (pH 8.0) [48]. The isolates were phylogenetically identified by amplifying and sequencing the 16S rRNA gene. Using universal primer sets, three sets of PCRs were performed. These primer sets amplify three partially overlapping regions of the 16S rRNA gene: 27F/785R (fragment F1; PCR product of approximately 750 bp), 341F/907R (F2; approximately 550 bp) and 785F/1492R (F3; approximately 700 bp). In a 50 µl PCR mix reaction, 0.02 U µl^−1^ (corresponding to 1.0 U per reaction) Taq DNA polymerase (Thermo-Fisher Scientific), 1×PCR buffer, 1.5 mM MgCl_2_, 0.2 mM dNTPs mix, 0.3 µM of each primer (SIGMA) and 1–3 µl template DNA were added. The PCR thermal protocol was as follows: (1) initial denaturation at 94 °C for 5 min; (2) 30 cycles of denaturation at 94 °C for 45 s; annealing at 52, 59 and 55 °C (F1, F2 and F3, respectively) for 1 min; and extension at 72 °C for 1 min; and (3) final extension at 72 °C for 10 min. The PCR products were purified using Illustra ExoProStar 1 Step (GE Life Sciences) and sequenced using forward and reverse primers via Sanger sequencing at the Bioscience Core Lab (KAUST). Electropherograms of the sequences were checked for quality, edited and assembled using Geneious v. 8.1.9 (Biomatters) to obtain almost full-length sequences (variable between 1300 and 1450 bp) of the 16S rRNA gene. The sequences obtained were then compared using the Basic Local Alignment Search Tool (blast) algorithm against the reference RNA sequences database (refseq_rna) of the National Centre for Biotechnology Information (NCBI) [49] and the arb Silva website (https://www.arb-silva.de) [50]. The sequences were submitted to NCBI under the accession numbers MW644816–MW644868. Following the procedures described by de Bruijn *et al*., enterobacterial repetitive intergenic consensus (ERIC) PCR was performed to examine the clonality of the selected strains [51].

The 16S rRNA sequence analyses of the 55 isolates revealed 13 unique bacterial strains after clustering at 99 % using the vsearch software [52]. These strains represented 9 different genera and 11 different species (Tables 1 and S1, Fig. S1a, available in the online version of this article). Among the 55 isolates, we detected two strains, R1DC25 and R1DC58, originating from DCs supplemented with sediment and leaf extracts, respectively, that had identical 16S rRNA gene sequences and ERIC PCR patterns (Figs S1b and S2, respectively) as well as <96 % similarity with a known species of the genus *Rhodoligotrophos *. (Table 1). On the basis of these results, we selected one of the two strains, R1DC25^T^ for further phylogenetic and physicochemical characterization.

**Table 1.** Taxonomic affiliation of isolated strains obtained using the diffusion chamber with sediment or leaf extracts. Detailed information for the 55 bacterial strains is reported in Table S1. The number of isolates belong to the same group (*i.e.*, clustering of 16S rRNA gene sequences at 99 % identity using the vsearch software [52]) is reported; it is followed by the taxonomy and accession number of the most closely related type strains and the range of percentage of identity. Among the isolates within each group, the representative bacterial strain is reported. The entry most closely related to the isolated strain described in this work is indicated in bold type.

Number of isolates	Most closely related type strain	Range of percentage identity	Reference isolated strain
11	* Isoptericola chiayiensis* 06182 M-1^T^ (NR_116696.1)	99.18–99.93	R1DC29
5	* Marinobacter adhaerens* HP15^T^ (NR_074765.1)	99.17–99.31	R1DC51
3	* Marinobacter salsuginis* SD-14B^T^ (NR_044044.1)	99.8–100	R1DC4
1	* Microbulbifer celer* ISL-39^T^ (NR_044243.1)	98.57	R1DC56
13	* Microbulbifer celer* ISL-39^T^ (NR_044243.1)	98.28–99.07	R1DC60
1	* Microbulbifer celer* ISL-39^T^ (NR_044243.1)	97.7	R1DC8
10	* Microbulbifer halophilus* YIM 91118^T^ (NR_044351.1)	98.08–98.34	R1DC16
1	* Muricauda aquimarina* SW-63^T^ (NR_042909.1)	98.62	R1DC39
1	* Pelagibaca bermudensis* HTCC 2601^T^ (NR_043611.1)	99.72	R1DC6
**2**	*** Rhodoligotrophos jinshengii* BUT-3^T^ (NR_134155.1**)	**94.43**	**R1DC25**
4	* Roseibium aggregatum* NBRC 16684^T^ (NR_113861.1)	99.01–99.27	R1DC21
1	* Saccharospirillum salsuginis* YIM-Y25^T^ (NR_044132.1)	97.09	R1DC57
2	* Salipiger mucosus* A3^T^ (NR_029116.1)	99.63	R1DC59

## Phylogeny based on the 16s rRNA gene and genome sequences

The genomic DNA of R1DC25^T^ was extracted using a Maxwell RSC Automated Nucleic Acid Purification and Maxwell RSC Cultured Cells DNA kits (Promega). Bacterial cultures were grown at 37 °C in MB for 48 h. The extracted DNA was quantified using Qubit dsDNA assay kits with a high sensitivity (Thermo-Fischer Scientific) and via electrophoresis on 1 % agarose gels and qualified using a Bioanalyzer 2100 (Agilent). Genomic DNA was sequenced using a PacBio RS2 sequencer (Pacific Biosciences) at the KAUST Bioscience Core Lab, and the reads were assembled using HGAP.3 workflow analysis [53]. The genome was then annotated using RAST, PROKKA and KEGG analysis for gene function prediction [54–57]. We submitted the complete genome of R1DC25^T^ under the GenBank accession number CP058214. The full-length 16S rRNA gene sequence of R1DC25^T^ was extracted and used for phylogenetic analysis; it was uploaded into GenBank under the accession number MT146881. A phylogenetic tree based on this 16S rRNA gene sequence was reconstructed by the neighbor-joining and maximum-likelihood method using the megax software (v10.1.8). To assess the significance of the generated tree, the topologies of the phylogenetic trees were evaluated using bootstrap analyses based on 1000 resamplings, as described previously [58, 59]. A multilocus sequence analysis (MLSA) or a phylogenomic tree of 120 concatenated single-copy genes obtained from the genome contig of the unclassified isolate was also included to illustrate the existing phylogenetic tree. The GTDB-Tk software (v 1.3) was used to analyse the phylogenetic diversity based on the best blast matches for the genes of these markers [60], and a bootstrap analysis of 1000 resamplings was used to evaluate the tree topology [61]. *In silico* digital DNA–DNA hybridization (dDDH) and blast-based average nucleotide identity (ANIb) scores of strains were calculated using the GGDC and JSpeciesWS software, respectively [61, 62], using default parameters. The percentage of conserved proteins (POCP) was also calculated as previously described [63].

**Fig. 1.**
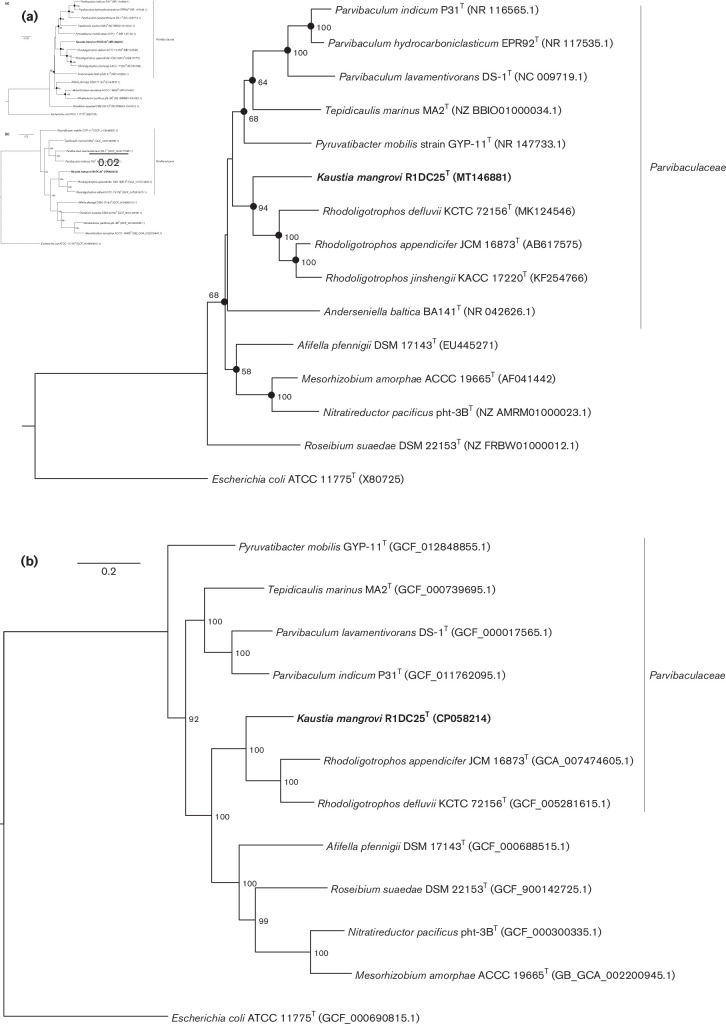
(**a**) Neighbor-joining phylogenetic tree based on 16S rRNA gene sequences showing the position of *Kaustia mangrovi* R1DC25^T^ (MT146881). Only bootstrap values (expressed as percentages of 1000 replications) of >50 % are shown at the branching points. Filled circles indicate branches that were also recovered using the maximum-likelihood method. *Escherichia coli* ATCC 11775^T^ (GenBank accession number X80725) was used as an outgroup. Bar, 0.020 substitutions per nucleotide position. The families of the different strains are shown on the right of the phylogenetic tree (**b**) Neighbor-joining phylogenomic tree using a MLSA concatenating 120 essential single-copy genes, highlighting the position of R1DC25^T^ relative to other closely related bacterial taxa within the order *Rhizobiales *. The tree was reconstructed using the software GTDB-Tk [60]. Numbers at the nodes designate bootstrap support values resulting from 1000 bootstrap replicates. Bar, 0.2 substitution per nucleotide position. Strains within family *Parvibaculaceae* are indicated by a vertical line.

Comparison of the 16S rRNA gene sequences indicated that the most closely related species to R1DC25^T^ were *Rhodoligotrophos appendicifer *, *Rhodoligotrophos jinshengii *,and *Rhodoligotrophos defluvii *, with sequence identities of 95.8, 95.3 and 94.5 %, respectively. The 16S rRNA gene-based neighbor-joining phylogenetic tree placed R1DC25^T^ away from the three species of the genus *Rhodoligotrophos* in the family *Rhodobiaceae* (Fig. 1a). Notably, the 16S rRNA gene identity values were only slightly above the threshold for the taxon boundaries of a novel genus (<95 % [64]); however, because R1DC25^T^ formed a separate branch and a novel clade that was different from the type strains of species of the genus *Rhodoligotrophos* (Fig. 1a), we investigated the phylogenetic placement of R1DC25^T^ in more detail. A phylogenomic reconstruction based on the MLSA of 120 concatenated conserved bacterial marker genes indicated that R1DC25^T^ clustered in a group distinct from the genus *Rhodoligotrophos* (Fig. 1b). When more strains within this family are sequenced, the differences between the *Kaustia* and *Rhodoligotrophos* genera will be clarified.

Compared with other identified species within the recently described family *Parvibaculaceae *, the ANIb was 66–69 % and dDDH was 18–19 % (Table 2)^46^. The standard ANIb criteria for genus discrimination is 45–65 % and that for species discrimination is 65–95 % [64] and the dDDH criterion is 70 % for novel species [61]. Accordingly, our data confirmed that R1DC25^T^ represents a novel species and possibly a novel genus within the family *Parvibaculaceae *. POCP has recently been described as valuable for the delineation of the prokaryotic genera (Table 2) [63]. The POCPs of the members of the genus *Rhodoligotrophos* were slightly above 50 %, which is the recommended threshold for genus delimitation. However, the thresholds for the definition of novel genera, *i.e.,* 16S rRNA gene identity, ANIb and POCP values [63, 64], are approximate, and both phylogenetic trees indicated that R1DC25^T^ is clearly separated from the members of the genus *Rhodoligotrophos *. Therefore, we propose that R1DC25^T^ represents a member of a novel genus. In addition, only two species were available within the genus *Rhodoligotrophos,* and all other members of the family *Parvibaculaceae* are phylogenetically distant from R1DC25^T^. The sequencing of more genomes in this group will enable us to elucidate the phylogenetic separation between R1DC25^T^ and members of the genus *Rhodoligotrophos *.

**Table 2.** Average nucleotide identity via blast (ANIb) and percentage of aligned sequences in brackets, using *in silico* digital DNA–DNA hybridization (dDDH) and percentage of conserved proteins (POCP) matrix of isolate *K. mangrovi* R1DC25^T^ with other representatives of the family *Parvibaculaceae *. The cut-off percentages to be assigned to the same species are ≥95 % and ≥70 % for ANIb and dDDH, respectively; the cut-off percentage to be assigned to the same genus is ≥50 % for POCP

Reference genome	ANIb (%)	dDDH (%)	POCP (%)
* Rhodoligotrophos appendicifer* JCM 16873^T^	69.3 [31.8]	18.7	53.0
* Rhodoligotrophos* sp*.* lm1^T^	69.2 [30.7]	18.7	54.7
* Tepidamorphus gemmatus* DSM 19345^T^	69.6 [27.7]	18.6	51.4
* Lutibaculum baratangense* AMV1^T^	69.2 [28.4]	18.5	49.8
* Amorphus coralli* DSM 19760^T^	69.0 [27.6]	18.5	50.6
* Mesorhizobium tamadayense* DSM 28320^T^	68.9 [25.5]	18.1	41.8
* Parvibaculum lavamentivorans* DS-1^T^	68.7 [20.0]	18.3	42.0
* Bauldia litoralis* ATCC 35022^T^	68.7 [24.9]	18.5	43.3
* Dichotomicrobium thermohalophilum* DSM 5002^T^	68.5 [21.9]	18.2	46.8
* Methyloceanibacter caenitepidi* Gela4^T^	68.5 [18.7]	18.3	39.5
* Mesorhizobium tianshanense* A-1BS^T^	68.5 [27.0]	18.0	41.7
* Mesorhizobium australicum* WSM2073^T^	68.3 [24.0]	18.2	42.1
* Oricola cellulosilytica* CC-AMH-0^T^	67.4 [20.1]	18.3	44.6
* Phyllobacterium endophyticum* PEPV15^T^	66.7 [19.5]	18.6	43.2

The genome length of R1DC25^T^ is 4.63 Mb, with a DNA G+C content of 67.3 mol% (Fig. S3). The genome was assembled in one contig comprising 4438 genes; of these, 4384 are protein-coding sequences and 54 are RNA-coding sequences. The genome includes two copies of the 16S rRNA gene (1522 bp) and 50 copies of tRNA. Of all protein-coding genes, 67 % were assigned a putative function, whereas the remaining genes were annotated as hypothetical proteins (Fig. S3). The presence of respiratory lipoquinones, cytochrome oxidases and membrane-bound electron transport chain-encoding genes confirmed that this strain is aerobic and can use oxygen as a terminal electron acceptor for respiration. However, this strain also possesses genes related to oxygen limitation (Table S2), such as the transporter system *fixLJK* [65], which allows resistance to the low-oxygen conditions of mangrove sediments [10, 66, 67]. Additionally, genome analysis confirmed the presence of genes encoding for osmoprotectant biosynthesis or transport (Table S2), such as the genes for proline (*proABC*, HW532_13700, HW532_17195 and HW532_137005), ectoine (*lysC*, HW532_10325) and betaine and choline (*betA*, HW532_01520) and for a glycine betaine/proline transport system (*proVWX*, HW532_05850–60), as well as genes associated with the synthesis of phytoene, a carotenoid precursor, via the nonmevalonate pathway. These compounds are used by R1DC25^T^ to adapt to the conditions of osmotic stress and the harsh mangrove environment [8, 10, 68], thus allowing survival in the salty sediments of the mangroves, where salinity can reach 15 % in summer [10]. The basic nature of the proteome, which has an average isoelectric point of 6.63, is similar to that of *Desulfohalobium retbaense* (6.5), a halophilic strain that uses the same osmoprotective mechanisms [69].

In addition to its capacity to survive the salinity and osmotic stress of the Red Sea mangrove sediments, R1DC25^T^ is able to survive the oligotrophic conditions of the mangrove forest [68]. This is partly because it possesses all the genes associated with the biosynthesis of amino acids and many transporters, such as *phoBDR*, *glnAGL* and *ntrYX*, that can be activated in case of nutrient limitation (Table S2). These transporters are important for survival under limited phosphate and nitrogen conditions [70–72]. However, *nifA*, which is responsible for nitrogen fixation, was not found, although it is normally regulated by the *ntrYX* transporter [71]. We also identified the presence of several genes involved in antibiotic resistance, particularly to beta-lactam and vancomycin, which may explain the resistance of the organism to a relatively large number of antibiotics as observed in *in vitro* tests conducted with Phenotype Microarray Biolog PM11 and PM12 plates described in the section on morphological, physiological and chemotaxonomic characterization.

As mangrove sediments, the environment of origin of R1DC25^T^, are dominated by plants and their widespread roots, we also investigated at the genome level the potential of this strain to interact with plants and promote plant growth. R1DC25^T^ does not possesses the complete pathways for flagellar biosynthesis, chemotaxis, root surface adhesion, quorum sensing or biofilm formation, which indicates that its lifestyle does not include a physical association with plant roots [73]. However, several genes encoding phytohormone production were identified, including those coding for 1-aminocyclopropane-1-carboxylic acid deaminase (HW532_02305) and auxin [indole acetic acid (IAA); *nthAB*, HW532_05650, HW532_05645] (Table S2). These results indicate the existence of a possible beneficial relationship between R1DC25^T^ and mangrove plants, which is mediated via a phytohormone homeostasis mechanism that positively affects mangroves, particularly during stressful conditions, such as drought and salinity, as previously observed in other natural and induced stress conditions [74–79]. Genes involved in biofertilization activity were also detected (Table S2). Among these were genes coding for the siderophore aerobactin (*iucABCD*, HW532_05380–95), which chelates iron and increases its availability to other macroorganisms and microorganisms [80–82] and competes as well for iron with possible plant pathogens [81]. Genome analysis revealed nitrate-reducing capacity involving *narGHI*, HW532_11500–15 (Table S2). This reaction is a major source of nitrogen for plant development and is also a limiting factor for mangrove growth in the oligotrophic conditions of the Red Sea coast [68]. These results strongly indicate that R1DC25^T^ is not directly or physically associated with the mangrove plants but can indirectly influence plant growth and fitness by acting as plant biofertilizer, growth biopromoter and pathogen biocontrol agent.

## Morphological, physiological and chemotaxonomic characterization

Cell morphology was examined using scanning electron microscopy [Teneo SEM (FEI)] at the Imaging Core Lab at KAUST. Motility was determined using semisolid 0.3 % MB agar. To determine the temperature range for growth, the strain was grown in 50 ml MB and incubated at temperatures of 10, 20, 30, 37, 40 and 50 °C (*n*=3 per temperature). OD_600_ was measured using a UV-1600PC spectrophotometer (VWR) every 12 h for 3 days. To evaluate the organism’s capacity to grow in the presence of salt, MB medium was prepared (recipe reported in the isolation and habitat section) but without salts. The salinity was <1 % as measured by a portable salinity refractometer; we considered this as 0 % salinity. Different concentrations of NaCl, 0–19 %, were added to the modified medium with 1 % as the incremental step and cultures were grown by incubating at 37 °C. OD_600_ of the cultures were measured using a UV-1600PC spectrophotometer at intervals of 12 h using non-inoculated media as the control. Further physiological and phenotypic characterization was performed using the Phenotype Microarray Biolog plates, and growth was investigated in the presence of different pH values (PM10) and sensitivity to antibiotics (PM11 and PM12 plates) using standard MB according to the manufacturers’ instructions. In case of PM9, wherein osmolytes and ions were present, bacterial cells were inoculated in the IF10 medium provided by Biolog, a rich medium with 0 % salinity. Carbon utilization was tested using the Phenotype Microarray Biolog plates (PM1 and PM2). To inoculate these plates, modified MB was prepared as previously reported but without adding peptone and yeast extract.

The oxidase activity of R1DC25^T^ was tested using oxidase strips (Sigma-Aldrich), and catalase activity was determined by bubble formation in 3 % (v/v) hydrogen peroxide solution [83]. Indole production was tested according to the method described by Bric *et al*. [84], and nitrate reducing ability was examined using nitrate reduction kits (Sigma) as per the manufacturers’ instructions. Nitrate broth medium was modified and supplemented with 4 % NaCl. Screening for enzyme activity was performed as described by Liu *et al*. [85] using MB agar plates to measure the activities of amylase, protease, lipase and cellulase. Furthermore, cellular fatty acids, respiratory quinones and polar lipids were analysed using the identification service laboratories of DSMZ (Braunschweig, Germany) for chemotaxonomic determination [86–88].

**Fig. 2.**
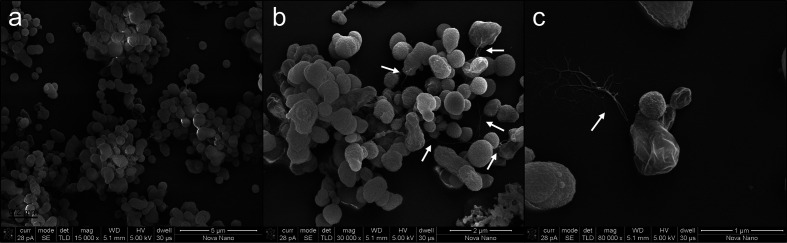
(**a–c**) Scanning electron micrographs of *Kaustia mangrovi* R1DC25^T^ cells grown on MB medium after 48 h of incubation at 37 °C. Bars' lengths are 5, 2 and 1 µm, respectively; arrows indicate the bacterial appendages outward from the surface of R1DC25^T^ cells.

The novel strain R1DC25^T^ was Gram-stain-negative, aerobic or facultatively anaerobic, non-motile and non-sporulating. Colonies were circular, with a diameter of 0.2–0.5 mm, smooth, shiny, with regular edges and of a creamy colour. Scanning electron microscopy of the colony-forming cells showed irregular coccoid cells 0.5–1 µm in diameter (Fig. 2a, b) with thin surface appendages (Fig. 2b, c) [89]. These appendages appeared to be implicated in surface adhesion (Fig. 2c) and possibly in bacterium–bacterium interactions during biofilm formation in the microbial mats [89].

R1DC25^T^ is a mesophilic bacterium that can grow between temperatures of a5 and 40 °C, with optimum temperature between 30 and 40 °C (Fig. S4a). The doubling time under optimum conditions at 37 °C was estimated as 5.2 h. It was able to grow in medium containing 2–14 % NaCl, with optimal growth at 3–5 % (Fig. S4b). These optimal conditions indicate that the strain is adapted to the salinity of the mangrove sediments in the Red Sea where no fresh water inputs occur and salinity can reach up to 15 % during summer [10]. The OD_600_ of R1DC25^T^ grown in MB with NaCl as the only salt was less than that of those grown using a mixture of salts (MgCl_2_, Na_2_SO_4_, CaCl_2_ and KCl) (Fig. S4b). This preference may indicate a strong dependence of R1DC25^T^ on sea salts. The results of PM9 Biolog plate experiments indicated that R1DC25^T^ is metabolically active. We measured the production of NADH in the presence of different osmolytes and ions, such as ectoine, betaine, proline, glycerol, sodium sulphate and sodium nitrate (Table S3), and the results revealed that the organism has the ability to osmoadapt under these conditions. Bacterial metabolism was inhibited in the presence of potassium chloride, ethylene glycol, sodium formate, urea, sodium lactate, sodium phosphate, sodium benzoate, ammonium sulphate and sodium nitrite (Table S3). R1DC25^T^ could grow at pH values ranging between 6 and 10, with optimum growth at pH 8.5 (Fig. S4c). These findings were in accordance with its environment of origin, where the pH of the sediments varied from 7.5 to 10 over the course of the year (Fusi, personal communication).

R1DC25^T^ is catalase-negative and oxidase-positive, and it can reduce nitrate to nitrite. The cells are negative for amylase, protease, lipase and cellulase and positive for the production of ammonia and IAA from tryptophan (Table 3). Of the 190 carbon sources available in the PM1 and PM2 plates, the strain showed active growth in the presence of 30 carbon sources (Table S4). Among these, 16 carbon sources showed strongly positive results, *i.e.,* twice that of the negative control, whereas the remaining 14 produced a weakly positive respiration with slower metabolism and growth (Table S4).

**Table 3.** General features and genomic and phenotypic characteristics of R1DC25^T^ and closely related members of the genus *Rhodoligotrophos
* Strains: 1, *K. mangrovi* R1DC25^T^ (data from this study); 2, *R. appendicifer* JCM 16873^T^ (data from [89] 90); 3, *R. jinshengii* BUT-3^T^ (data from [91]); 4, *R. defluvii* lm1^T^ (data from [85] 60). +, Positive; −, negative; na, not available.

Characteristics	1	2	3	4
**General features**				
Isolation source	Mangrove sediments	Freshwater	Activated sludge	Activated sludge
Cell morphology	Irregular coccoid	Irregular coccoid	Ovoid	Short rod
Appendaged cells	+	+	−	−
Colony colour	Creamy	Orange-red	Red	Red
Motility	Nonmotile	Nonmotile	Nonmotile	Nonmotile
Biotic relationship	Free living	Free living	Free living	Free living
Temperature range (optimum, °C)	15–40 (30–40)	5–35 (30)	15–40 (30)	15–45 (40)
NaCl% range (optimum, %)	2–14 (3–5)	0–5 (0.5–1)	0–7 (1.5–3)	0–4 (1–2)
pH range (optimum)	6–10 (8.5)	6–9 (7)	5–10 (7)	4–10 (8)
**Genome features**				
Genome size (Mb)	4.63	5.49	na	5.49
DNA G+C content (mol%)	67.3	61.1	67.7	64.4
**Membrane features**				
Predominant ubiquinone	Q-10	Q-9	Q-10	Q-10
Polar lipids	PG, PC, DPG, AL, L	PE, PG, PC, DPG, AL, GL, L	PE, PG, PC, DPG, AL, GL, L	PE, PG, PC, DPG, AL, GL, L
**Enzymatic activity**				
Indole production	+	na	–	–
Nitrate reduction	+	–	–	na
Oxidase reaction	+	+	–	–
Catalase reaction	–	+	–	+
**Substrate utilization**				
l-arabinose	+	+	+	na
d-glucose	–	+	+	+
d-mannose	–	–	+	–
d-mannitol	–	–	+	–
Maltose	–	–	+	–
Sucrose	–	–	+	–
Rhamnose	–	+	–	–
Pyruvate	+	na	na	+

AL, Alabaster; DPG, Diphosphotidyglycerol; GL, Glycolipid; L, Lipid; PC, Phosphatidylcholines; PE, Phosphatidylethanolamine; PG, Phosphatidylglycerol.

The predominant respiratory quinone of R1DC25^T^ was ubiquinone Q-10 (100%) as seen in many members of the class *Alphaproteobacteria* [89]. The cellular fatty acids of R1DC25^T^ are composed of C_19 : 0_cyclo *ω*8*c*, a combination of C_18 : 1_ *ω*7*c* and/or C_18 : 1_ *ω*6*c* and C_16 : 0_ (Table S5). This was consistent with the results of the phylogenetic analysis and confirmed the separate taxonomic status of the novel isolate [90]. R1DC25^T^ was clearly distinguished from other species of the genus *Rhodoligotrophos* due to the predominance of a combination of unsaturated chain fatty acids, cyclo-C_19 : 0_ *ω*8*c* (47.54 %), and straight chain saturated fatty acids, C_16 : 0_ (15.54 %) with the presence of C_18 : 1_ *ω7c* and/or C_18 : 1_ *ω*6*c* (summed feature 8; 20.71 %), which have not been detected in members of the genus *Rhodoligotrophos *. The proportion of cyclo-C_19 : 0_ *ω*8*c* was higher in R1DC25^T^ than in other related species, and, in contrast to the related species, the methylated fatty acids 11 methyl-C_18 : 1_ *ω*7*c* and 10 methyl-C_19 : 0_ were present in R1DC25^T^ and C_18 : 1_ *ω*9*c* and branched saturated chain fatty acids were absent (Table S5). The cellular fatty acid composition may change depending on the medium in which cells have been grown, and the comparison of the fatty acids of R1DC25^T^ with published descriptions of fatty acids of the strains of members of the genus *Rhodoligotrophos* should be taken with caution; however, the differences observed among the fatty acids (31 –49% of fatty acids) support the classification of R1DC25^T^ as a representative of a novel genus. The polar lipids are phosphatidylglycerol, phosphatidylcholine, diphosphatidylglycerol, several distinct aminolipids, three unidentified aminolipids and two unidentified lipids (Table 3). This profile was different from those of the other species of the genus *Rhodoligotrophos* because of the absence of phosphatidylethanolamine, glycolipid and five unknown lipids.

Overall, chemotaxonomic characterization indicated that R1DC25^T^ is distinct from the most closely related species within the genus *Rhodoligotrophos* (Table 3), and it can be also differentiated from the phylogenetically closely related genera within the family *Rhodobiaceae* (Table S6), supporting its classification as a member of a novel genus.

On the basis of data obtained from a polyphasic approach, strain R1DC25^T^ represents a novel species of the novel genus in the *Rhodobiaceae *, for which the name *Kaustia mangrovi* gen. nov., sp. nov. is proposed.

## Description of *Kaustia* gen. nov.

*Kaustia* [Kaus′ti.a. N.L. fem. n. *Kaustia* subjective name derived from the abbreviation KAUST (King Abdullah University of Science and Technology)].

The bacterial cells are Gram-stain-negative, aerobic/facultatively anaerobic, moderately halophilic, mesophilic, non-motile, non-sporulating, catalase-negative, oxidase-positive and nitrate-reduction-positive. The cellular fatty acids included significant amounts (>5%) of C_19 : 0_cyclo *ω*8*c*, a combination of C_18 : 1_ *ω*7*c* and/or C_18 : 1_ *ω*6*c* and C_16 : 0_. Ubiquinone Q-10 (100%) is the major respiratory quinone. The polar lipid composition is dominated by phosphatidylglycerol, phosphatidylcholine and diphosphatidylglycerol, as well as several distinct aminolipids and lipids.

The type species is *Kaustia mangrovi*.

## Description of *Kaustia mangrovi* sp. nov.

*Kaustia mangrovi* (man.gro′vi. N.L. gen. n. *mangrovi* of a mangrove).

Colonies are circular and creamy in colour and measure 0.8 µm in diameter. Growth occurs at 2–14 % NaCl (optimum, 3–5 %), 15–40 °C (optimum, 30–40 °C) and a pH of 6.5–10 (optimum, 8.5). Can grow in the presence of several osmolytes and ions at different concentrations. Cells are positive for the production of indole and ammonia from tryptophan, but are negative for amylase, protease, lipase and cellulase activity. The following substrates are used: l-arabinose, d-arabinose, d-glucosamine, d-saccharinic acid, dihydroxy acetone, l-alaninamide, l-alanine, l-asparagine, l-glutamic acid, l-glutamine, l-ornithine, l-proline, mucic acid, oxalomalic acid, pyruvic acid, 5-keto-d-gluconic acid and pyruvate.

The type strain is R1DC25^T^ (=KCTC 72348^T^=JCM 33619^T^=NCCB 100699^T^) isolated from the mangrove sediments on the coast of the Red Sea in KAUST (Thuwal, Saudi Arabia). The genome of the type strain has a size of 4.63 Mb and a DNA G+C content of 67.3 mol%. The GenBank accession number for the 16S rRNA gene sequence of strain R1DC9^T^ extracted from the genome DNA sequence is MT146883. The whole-genome shotgun sequence project was deposited at DDBJ/EMBL/GenBank under the following accession identification: CP028923.1.

## Supplementary Data

Supplementary material 1
