# Corrigendum: *Kaustia mangrovi* gen. nov., sp. nov. isolated from Red Sea mangrove sediments belongs to the recently proposed *Parvibaculaceae* family within the order Rhizobiales

**DOI:** 10.1099/ijsem.0.004937

**Published:** 2021-08-19

**Authors:** Fatmah O. Sefrji, Ramona Marasco, Grégoire Michoud, Kholoud A. Seferji, Giuseppe Merlino, Daniele Daffonchio

**Affiliations:** ^1^​ Biological and Environmental Sciences and Engineering Division (BESE), Red Sea Research Center (RSRC), King Abdullah University of Science and Technology (KAUST), Thuwal, Saudi Arabia

In the published version of this article there were errors in the abstract, the first page footnote and the section entitled ‘Description of *Kaustia mangrovi* sp. nov.’ in the protologue.

In the published version of the abstract the explanation of the origin of the genus name *Kaustia* between parentheses in the last two lines was incorrect. The text between the parentheses should have read as follows:

‘(* Kaustia *, arbitrary name derived from the abbreviation KAUST for King Abdullah University of Science and Technology; *mangrovi*, of a mangrove).’

Additionally, the following sentence in the first page footnote included strain name and DNA sequence accession numbers that were incorrect and should have been omitted: ‘The GenBank accession number for the 16S rRNA gene sequence of strain R1DC9^T^ extracted from the genome DNA sequence is MT146883. The whole-genome shotgun sequence project was deposited at DDBJ/EMBL/GenBank under the following accession identification: CP028923.1.’

In the published version of this article the following errors were present in the ‘Description of *Kaustia mangrovi* sp. nov.’ in the protologue; a non-type strain, R1DC9 was erroneously annotated as a type strain in the protologue of the type species of the genus, the genome sequence CP028923.1 and the 16S rRNA gene sequence given in the species protologue were incorrect.

The descriptions of the genus *Kaustia* and the type species *Kaustia mangrove* should have read as follows:

## ‘Description of *Kaustia* gen. nov.

*Kaustia* (Kaus´ti.a. N.L. fem. n. *Kaustia* arbitrary name derived from the abbreviation KAUST [King Abdullah University of Science and Technology]).

The bacterial cells are Gram-stain-negative, aerobic/facultatively anaerobic, moderately halophilic, mesophilic, non-motile, non-sporulating, catalase negative, and oxidase and nitrate-reduction positive. The cellular fatty acids included significant amounts (>5 %) of C_19 : 0_cyclo *ω*8c, a combination of C_18 : 1_ *ω*7c and/or C_18 : 1_ *ω*6c, and C_16 : 0_. Ubiquinone Q-10 (100 %) is the major respiratory quinone. The polar lipid composition is dominated by phosphatidylglycerol, phosphatidylcholine, diphosphatidylglycerol, as well as several distinct aminolipids and lipids.

The type species is *Kaustia mangrovi *.

## Description of *Kaustia mangrovi* sp. nov.

*Kaustia mangrovi* (man.gro´vi. N.L. gen. n. *mangrovi* of a mangrove).

Colonies are circular and creamy in colour, and measure 0.8 mm in diameter. Growth occurs at 2–14% NaCl (optimum, 3–5 %), 15–40 °C (optimum, 30–40 °C), and a pH of 6.5–10 (optimum, 8.5). The strain can grow in the presence of several osmolytes and ions at different concentrations. Cells are positive for the production of indole from tryptophan and ammonia, but are negative for amylase, protease, lipase, and cellulase activity. The following substrates are used: l-arabinose, d-arabinose, d-glucosamine, d-saccharic acid, dihydroxy acetone, l-alaninamide, l-alanine, l-asparagine, l-glutamic acid, l-glutamine, l-ornithine, l-proline, mucic acid, oxalomalic acid, pyruvic acid and 5-keto-d-gluconic acid.

The type strain is R1DC25^T^ (=KCTC 72348^T^=JCM 33619^T^=NCCB 100699^T^) isolated from the mangrove sediments on the coast of the Red Sea in KAUST (Thuwal, Saudi Arabia). The genome of the strain has a size of 4.63 Mb and a DNA G+C content of 67.3 mol%. The GenBank accession number for the 16S rRNA gene sequence of strain R1DC25^T^ extracted from the genome DNA sequence is MT146881. The whole-genome shotgun sequence project was deposited at DDBJ/EMBL/GenBank under the following accession identification: CP058214.’

The authors apologise for any inconvenience caused.

