# Supplementary material for: Kaustia mangrovi gen. nov., sp. nov. isolated from Red Sea mangrove sediments belongs to the recently proposed Parvibaculaceae family within the order Rhizobiales
Source: Int J Syst Evol Microbiol. 2021 May 17;71(5):004806. doi: 10.1099/ijsem.0.004806 (PMC8289202; doi:10.1099/ijsem.0.004806)
Supplement: Supplementary material 1 [file ijsem-71-4806-s001.pdf]

Supplementary Material to the manuscript

Kaustia mangrovi **gen. nov., sp. nov. isolated from Red Sea mangrove sediments belongs to the recently proposed** ***Parvibaculaceae*** **family within the order Rhizobiales**

Fatmah O. Sefrji[1], Ramona Marasco[1], Grégoire Michoud[1], Kholoud Seferji[1], Giuseppe Merlino[1], Daniele Daffonchio[1#]

[1]Red Sea Research Center (RSRC), King Abdullah University of Science and Technology (KAUST), Thuwal, Saudi Arabia

[#]Corresponding authors: Daniele Daffonchio, King Abdullah University of Science and Technology (KAUST), Red Sea Research Center (RSRC), Thuwal, Saudi Arabia. Phone: +966(2)8082884; E-mail: daniele.daffonchio@kaust.edu.sa

Supplementary Table S1. List of bacteria isolated using the first round of diffusion chambers incubation. Percentage of identity and closest type strains are indicated.

Isolates	Extract	% identity	Closest type strain	Reference strain
R1DC10	Sediment	98.15	*Microbulbifer halophilus* YIM 91118^T (NR_044351.1)	**R1DC16**
R1DC11	Sediment	98.15		
R1DC13	Sediment	98.28		
R1DC14	Sediment	98.32		
R1DC15	Sediment	98.08		
R1DC16	Sediment	98.09		
R1DC17	Sediment	98.27		
R1DC19	Sediment	98.34		
R1DC20	Sediment	98.27		
R1DC24	Sediment	98.33		
R1DC21	Sediment	99.15	*Roseibium aggregatum* NBRC 16684T (NR_113861.1)	**R1DC21**
R1DC22	Sediment	99.22		
R1DC23	Sediment	99.01		
R1DC46	Leaves	99.27		
R1DC58	Leaves	94.43	*Rhodoligotrophos jinshengii* BUT-3^T (NR_134155.1)	**R1DC25**
R1DC25	Sediment	94.43		
R1DC28	Sediment	99.18	*Isoptericola chiayiensis* 06182M-1^T (NR_116696.1)	**R1DC29**
R1DC29	Sediment	99.25		
R1DC31	Leaves	99.93		
R1DC32	Leaves	99.93		
R1DC33	Leaves	99.93		
R1DC35	Leaves	99.93		
R1DC37	Leaves	99.86		
R1DC38	Leaves	99.93		
R1DC40	Leaves	99.93		
R1DC44	Leaves	99.93		
R1DC50	Leaves	99.86		
R1DC39	Leaves	98.62	*Muricauda aquimarina* SW-63^T (NR_042909.1)	**R1DC39**
R1DC34	Leaves	100.00	*Marinobacter salsuginis* SD-14B ^{T} (NR_044044.1)	**R1DC4**
R1DC4	Sediment	99.80		
R1DC43	Leaves	100.00		
R1DC45	Leaves	99.31	*Marinobacter adhaerens* HP15 ^{T} (NR_074765.1)	**R1DC51**
R1DC48	Leaves	99.17		
R1DC49	Leaves	99.31		
R1DC51	Leaves	99.31		
R1DC55	Leaves	99.30		
R1DC56	Leaves	98.57	*Microbulbifer celer* ISL-39 ^{T} (NR_044243.1)	**R1DC56**
R1DC57	Leaves	97.09	*Saccharospirillum salsuginis* YIM-Y25 ^{T} (NR_044132.1)	**R1DC57**
R1DC54	Leaves	99.63	*Salipiger mucosus* A3 ^{T} (NR_029116.1)	**R1DC59**
R1DC59	Leaves	99.63		
R1DC6	Sediment	99.72	*Pelagibaca bermudensis* HTCC260 ^{T} (NR_043611.1)	**R1DC6**
R1DC1	Sediment	98.29	Microbulbifer celer ISL-39 ^{T} (NR_044243.1)	**R1DC60**
R1DC12	Sediment	98.39		
R1DC18	Sediment	98.43		
R1DC2	Sediment	98.29		
R1DC26	Sediment	98.44		
R1DC27	Sediment	98.43		
R1DC3	Sediment	98.43		
R1DC36	Leaves	98.43		
R1DC42	Leaves	99.07		
R1DC47	Leaves	98.37		
R1DC5	Sediment	98.43		
R1DC60	Leaves	98.44		
R1DC7	Sediment	98.28		
R1DC8	Sediment	97.70	*Microbulbifer celer* ISL-39 ^{T} (NR_044243.1)	**R1DC8**

Supplementary Table S2. Genes associated with PGP traits (biopromotion and biofertilization), nutrients limitation and adaptative traits in *K. mangrovi* R1DC25^T.

**Function**	**Gene name**	**Best hit (HW532_)**
**Biopromotion**		
ACC deaminase	*accD*	02305
Auxin production	*nthA, nthB*	05650, 05645
**Biofertilization**		
Nitrogen metabolism	*glnA, glnG, glnL, ntrY, ntrX, narG, narH, narI*	06480, 01845, 01850, 01835, 01840, 11500, 11505, 11510, 11515
Phosphate limitation	*phoBD*	13245, 11230
Siderophore	*iucA, iucB, iucC, iucD*	05395, 05390, 05385, 05380
**Adaptative traits**		
Aerobactin production	*iucA, iucB, iucC, iucD*	05380, 05385, 05390, 05395
Oxygen limitation	*fixJ, fixK, fixL*	08040, 08030, 08045
Osmoprotectant	*proA, proB, proC, lysC, betA, proV, proW, proX*	13700, 17195, 137005, 10325, 01520, 05850; 05855, 05860
Phytoene (carotenoid precursor)	*crtB*	17965

Supplementary Table S3. Metabolic profiling of strain R1DC25^T on Biolog PM9 microplate. Active growth (measured as NADH production during cell respiration) of strain R1DC25^T was reported as positive, ++; weakly positive, +; negative, -. In case a substrate is reported at different concentration, range in which the strain can growth (+ and ++) or not (-) is indicated.

**Substrate**	**Growth***	**Substrate**	**Growth***
NaCl 1-8%	++	NaCl 6% + Glycerol	++
NaCl 9-10%	+	NaCl 6% + Trehalose	++
NaCl 6% + Betaine	++	NaCl 6% + Trimethylamine-N-oxide	++
NaCl 6% + N-N Dimethyl glycine	++	NaCl 6% + Trimethylamine	++
NaCl 6% + Sarcosine	++	NaCl 6% + Octopine	++
NaCl 6% + Dimethyl sulphonyl propionate	++	NaCl 6% + Trigonelline	+
NaCl 6% + MOPS	++	Potassium chloride 3	+
NaCl 6% + Ectoine	++	Potassium chloride 4-6%	++
NaCl 6% + Choline	++	Sodium sulphate 2-5%	++
NaCl 6% + Phosphoryl choline	++	Ethylene glycol 5-20%	-
NaCl 6% + Creatine	++	Sodium formate 1-6%	-
NaCl 6% + Creatinine	++	Urea 2-7%	-
NaCl 6% + L-Carnitine	+	Sodium Lactate 1-12%	-
NaCl 6% + KCl	++	Sodium Phosphate pH 7 20-200 mM	-
NaCl 6% + L-proline	++	Sodium Benzoate pH 5.2 20-200 mM	-
NaCl 6% + N-Acethyl L-glutamine	++	Ammonium sulphate pH 8 10-100 mM	-
NaCl 6% + ß-Glutamic acid	++	Sodium Nitrate 10-100 mM	+
NaCl 6% + γ-Amino-n-butyric acid	++	Sodium Nitrite 10	+
NaCl 6% + Glutathione	++	Sodium Nitrite 20-100 mM	-

*Measured as reduction a tetrazolium dye consequently to cell respiration (*i.e.*, NADH production)

Supplementary Table S4. Carbon sources utilized by *K. mangrovi* R1DC25^T; capacity of R1DC25^T to utilize carbon sources for growth was measured as respiration activity (NADH production) and reported as ++ and +, respectively positive and weakly positive growth; the remaining carbon sources of the Biolog PM1 and 2 not reported in the present table were considered negative (*i.e.*, R1DC25^T is not able to utilize them).

**Carbon source**	**Growth***	**Carbon source**	**Growth***
D-Arabinose	++	Acetic Acid	+
D-Glucosamine	++	Butyric Acid	+
D-Saccharin Acid	++	D, L-Carnitine	+
Dihydroxy Acetone	++	Glycine	+
L-Alaninamide	++	Hydroxy-L- Proline	+
L-Alanine	++	L-Arabinose	+
L-Asparagine	++	L-Homoserine	+
L-Glutamic Acid	++	L-Isoleucine	+
L-Glutamine	++	L-Lactic Acid	+
L-Ornithine	++	L-Lysine	+
L-Proline	++	L-Phenylalanin	+
Mucic Acid	++	Mono Methyl Succinate	+
Oxalomalic Acid	++	Succinate	+
Pyruvic Acid	++	β-Hydroxy Butyric Acid	+
α-Keto-Glutaric Acid	++		
5-Keto-D-Gluconic Acid	++		

*Measured as reduction a tetrazolium dye consequently to cell respiration (*i.e.*, NADH production)

Supplementary Table S5. Cellular fatty acid composition (%) of strain R1DC25^T and of the members of the genus *Rhodoligotrophos*. Strains: 1, *K. mangrovi* R1DC25^T (data from this study); 2, *R. appendicifer* JCM 16873^T (data from Fukuda *et al*., [4]); 3, *R. jinshengii* BUT-3^T (data from Deng *et al*., [5]); 4, *R. defluvii* lm1^T (data from Liu *et al*., [6]). Star *: summed features (SF) are groups of two or three fatty acids that could not be separated by GLC with the MIDI system. SF3 comprises iso-$C_{15:0}$ 2-OH and/or $C_{16:1}$ *ω*7c and/or $C_{16:1}$ *ω*6c. SF8 comprises $C_{18:1}$ *ω*7c and/or $C_{18:1}$ *ω*6c. Values shown are percentages of total fatty acids with major components highlighted in bold (> 5%); -, not detected.

Fatty acid type	Fatty acid composition	1	2	3	4
**Saturated straight chain**	$C_{12:0}$	-		0.4	0.5
	$C_{14:0}$	-		1.0	0.7
	$C_{16:0}$	**15.54**	**22.6**	**14.3**	**17.0**
	$C_{17:0}$	1.00			
	$C_{18:0}$	2.30		2.0	1.6
**Unsaturated branch chain**	anteiso-$C_{15:1}$ A	-		0.8	
	$C_{16:1}$ *ω*7c	-	**9.5**		
	$C_{17:1}$ *ω*8c				0.1
	$C_{18:1}$ *ω*5c				0.2
	$C_{18:1}$ *ω*7c	-	**12.1**	**19.1**	**29.0**
	$C_{18:1}$ *ω*9c	-	**14.1**	1.8	
	$C_{19:0}$ cyclo *ω8c*	**47.54**		**38.2**	**44.2**
	$C_{20:2}$ *ω*6c9	2.34			
**Saturated branch chain**	iso-$C_{14:0}$	-		0.3	
	anteiso-$C_{15:0}$	-		**6.6**	0.2
	iso-$C_{15:0}$	-		**5.3**	0.6
	iso-$C_{16:0}$	-		0.7	0.1
	iso-$C_{17:0}$	-		0.8	
	anteiso-$C_{17:0}$	-		1.5	0.1
	$C_{17:0}$ cyclo	-	**9.5**	1.4	0.5
**Hydroxylated**	$C_{14:0}$ 3-OH	-	**7.8**		
	$C_{16:0}$ 2-OH	1.01		0.4	0.2
	$C_{18:0}$ 3-OH	2.62			
	$C_{18:1}$ 2-OH	1.66		1.0	
**Methylated**	11 methyl-$C_{18:1}$ *ω7c*	2.79			
	10 methyl-$C_{19:0}$	1.81			
**SF3***		0.69		1.8	3.3
**SF8***		**20.57**			
**Unknown** 14.959		0.66			

Supplementary Table S6. Differential characteristics of strain R1DC25^T and the phylogenetically closely related genera in the family *Rhodobiaceae*. Taxa: 1, strain R1DC25^T (this study); 2, *Anderseniella* (Brettar *et al*., 2007 [1]); 3. *Parvibaculum* (Takeuchi *et al*., 2015 [2]); 4, *Pyruvatibacter* (Wang *et a*l., 2016 [3]); 5, *Rhodoligotrophos* (Fukuda *et al*., 2012 ; Deng *et al*., 2014 ; Liu *et al*., 2019 [4–6]); 6, *Tepidicaulis* (Takeuchi *et al*., 2015 [2]). NA: no data.

**Characteristic**	**1**	**2**	**3**	**4**	**5**	**6**
Motility	non-motile	non-motile	motile	motile	non-motile	non-motile
Temperature optimum (°C)	30-40	25-30	20–40	30	30-40	42
NaCl% optimum (%)	3-5	1.5 -3	2.5	1 - 3	0.5-1	3
pH optimum	8.5	7–7.5	7.5	7 - 8	7-8	8
DNA G+C content (mol%)	67.3	61.2	60.7	63	60.7–64.5	60.7
Major fatty acids	$C_{19:0}$ cyclo	$C_{18:1}$, $C_{19:0}$ cyclo	$C_{18:1}$, $C_{19:0}$ cyclo	$C_{18:1}$	$C_{16:0}$, $C_{18:1}$	$C_{18:1}$, $C_{16:0}$
Predominant ubiquinone	Q-10	NA	Q-10-Q-11	Q-10	Q-9-Q10	Q-10

Supplementary Figure S1. (**a**) Phylogenetic tree of isolates obtained from the diffusion chamber cultivation. Isolates were clustered based on their sequence identity (99%) and only the reference sequences are shown (see Table 1). (**b**) Comparison of the 16S rRNA sequences of the two strains.

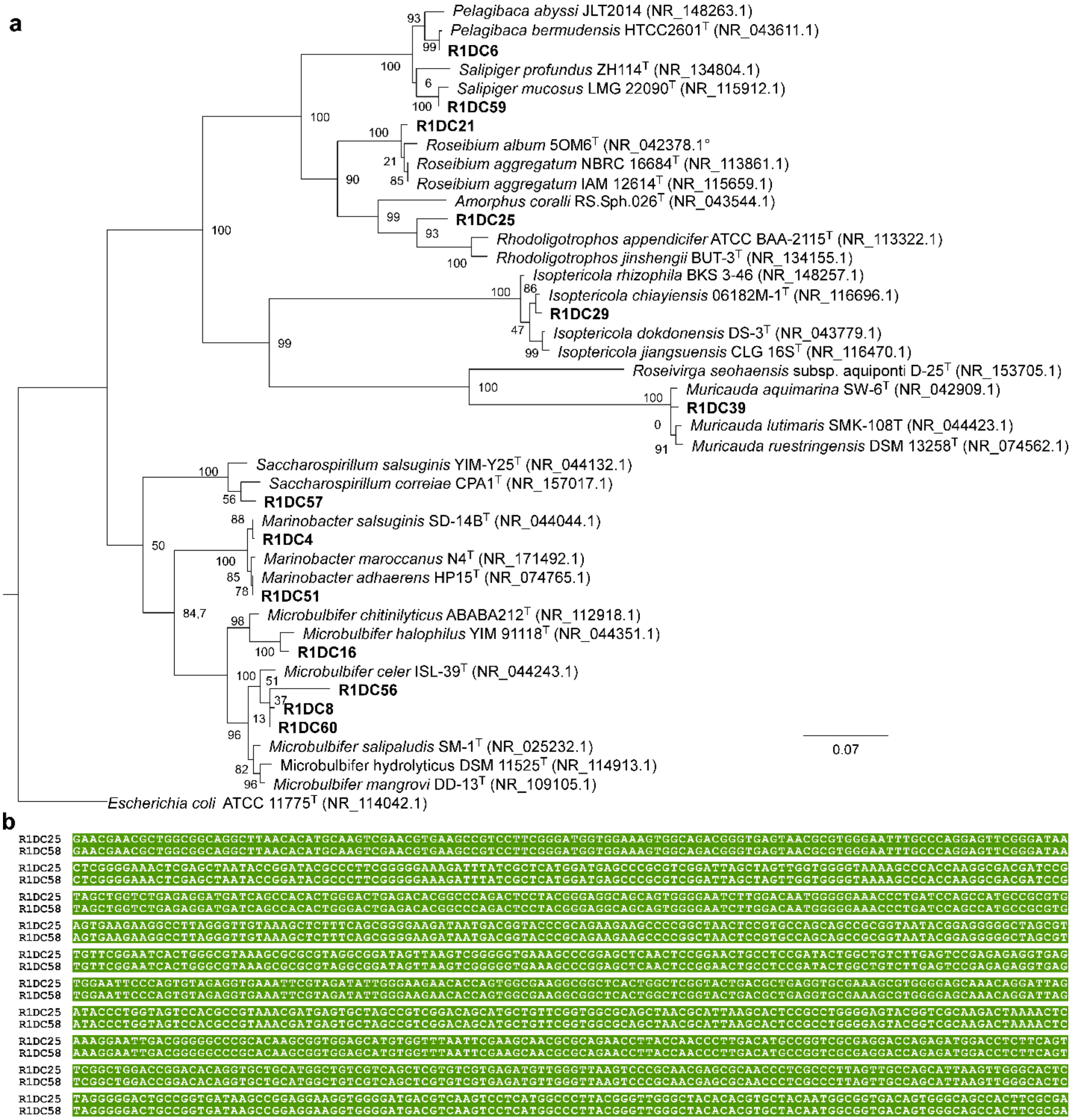

Supplementary Figure S2. ERIC PCR Fingerprinting patterns of the PCR products generated by using the ERIC primers for strains R1DC25 (K1) and R1DC58 (K2); ERIC PCR products are visualized by bioanalyzer Agilent 2100, using high sensitivity dsDNA kit.

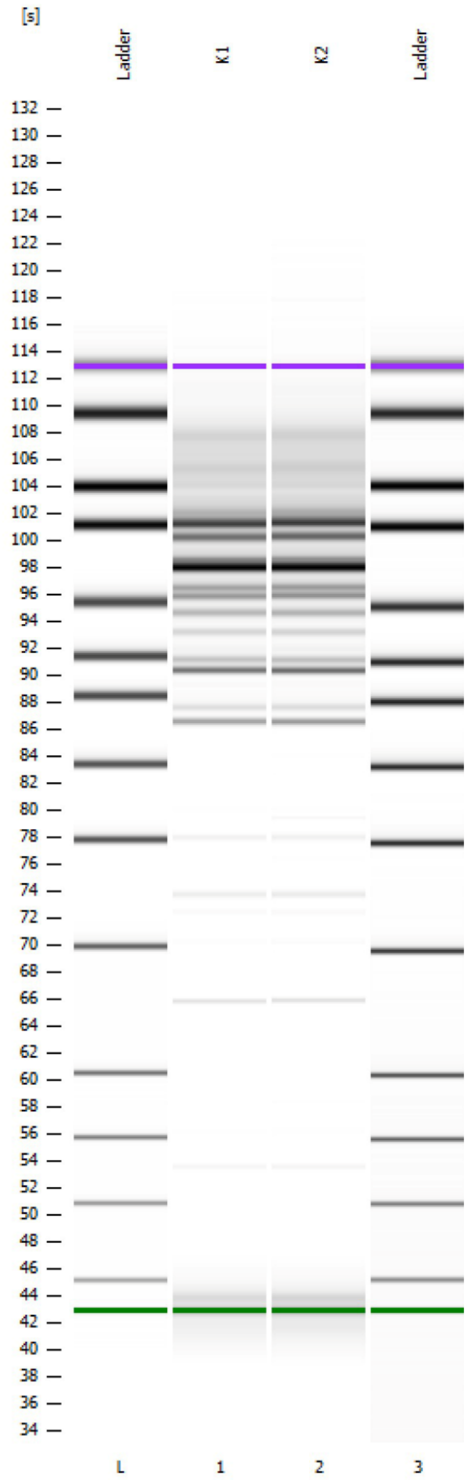

Supplementary Figure S3. Graphical circular map of the chromosome and genome features of *K. mangrovi* R1DC25^T.

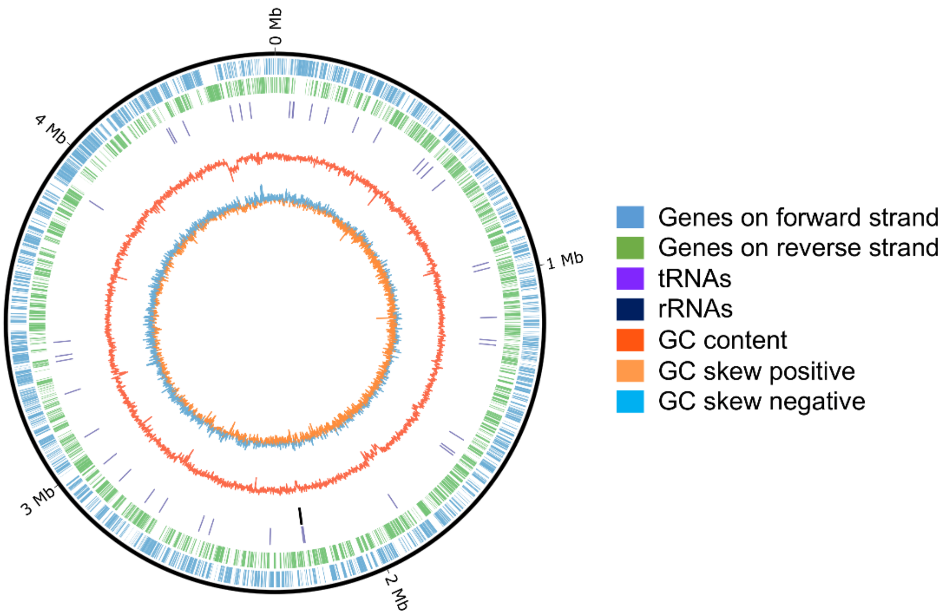

Supplementary Figure S4. Strain *K. mangrovi* R1DC25^T growth at (**a**) different temperatures, in presence of (**b**) increasing concentrations of salt (NaCl) and MB medium, and (**c**) different pH. Values are expressed as average of optical density (OD_{600}, n=3) in **a** and **b**, and as average of cell respiration (NADH production; n=2) in **c**.

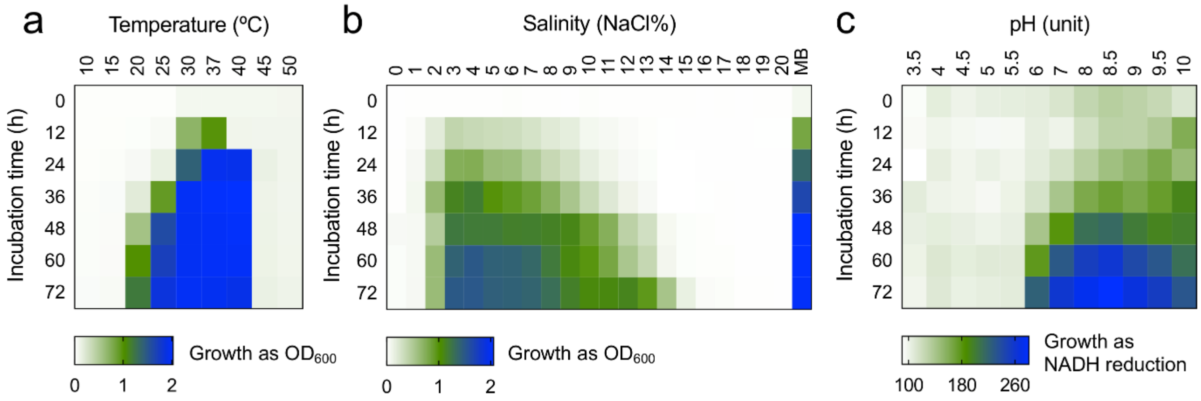

References

1. **Brettar I, Christen R, Bötel J, Lünsdorf H, Höfle MG**. Anderseniella baltica gen. nov., sp. nov., a novel marine bacterium of the Alphaproteobacteria isolated from sediment in the central Baltic Sea. *Int J Syst Evol Microbiol* 2007;57:2399–2405.
2. **Takeuchi M, Yamagishi T, Kamagata Y, Oshima K, Hattori M, *et al.*** Tepidicaulis marinus gen. nov., sp. nov., a marine bacterium that reduces nitrate to nitrous oxide under strictly microaerobic conditions. *Int J Syst Evol Microbiol* 2015;65:1749–1754.
3. **Wang G, Tang M, Wu H, Dai S, Li T, *et al.*** Pyruvatibacter mobilis gen. nov., sp. nov., a marine bacterium from the culture broth of Picochlorum sp. 122. *Int J Syst Evol Microbiol* 2016;66:184–188.
4. **Fukuda W, Yamada K, Miyoshi Y, Okuno H, Atomi H, *et al.*** Rhodoligotrophos appendicifer gen. nov., sp. nov., an appendaged bacterium isolated from a freshwater Antarctic lake. *Int J Syst Evol Microbiol* 2012;62:1945–1950.
5. **Deng SK, Chen GQ, Chen Q, Cai S, Yao L, *et al.*** Rhodoligotrophos jinshengii sp. nov., isolated from activated sludge. *Int J Syst Evol Microbiol* 2014;64:3325–3330.
6. **Liu Y-L, Meng D, Li R-R, Gu P-F, Fan X-Y, *et al.*** Rhodoligotrophos defluvii sp. nov., isolated from activated sludge. *Int J Syst Evol Microbiol* 2019;3830–3836.
